# Reporting guidelines for clinical trials of artificial intelligence interventions: the SPIRIT-AI and CONSORT-AI guidelines

**DOI:** 10.1186/s13063-020-04951-6

**Published:** 2021-01-06

**Authors:** Hussein Ibrahim, Xiaoxuan Liu, Samantha Cruz Rivera, David Moher, An-Wen Chan, Matthew R. Sydes, Melanie J. Calvert, Alastair K. Denniston

**Affiliations:** 1 Academic Unit of Ophthalmology, Institute of Inflammation and Ageing, College of Medical and Dental Sciences, University of Birmingham, Birmingham, B15 2TT UK; 2 University Hospitals Birmingham NHS Foundation Trust, Birmingham, UK; 3 Centre for Regulatory Science and Innovation, Birmingham Health Partners, Birmingham, UK; 4 Moorfields Eye Hospital NHS Foundation Trust, London, UK; 5 Health Data Research UK, London, UK; 6 Centre for Patient Reported Outcomes Research, Institute of Applied Health Research, University of Birmingham, Birmingham, UK; 7 Institute of Applied Health Research, University of Birmingham, Birmingham, UK; 8 Centre for Journalology, Clinical Epidemiology Program, Ottawa Hospital Research Institute, Ottawa, Canada; 9 School of Epidemiology and Public Health, Faculty of Medicine, University of Ottawa, Ottawa, Canada; 10 Department of Medicine, Women’s College Research Institute, Women’s College Hospital, University of Toronto, Toronto, Ontario Canada; 11 Medical Research Council Clinical Trials Unit at University College London, Institute of Clinical Trials and Methodology, University College London, London, UK; 12 National Institute for Health Research Birmingham Biomedical Research Centre, University of Birmingham and University Hospitals Birmingham NHS Foundation Trust, Birmingham, UK; 13 National Institute for Health Research Applied Research Collaborative West Midlands, Coventry, UK; 14 National Institute for Health Research Surgical Reconstruction and Microbiology Centre, University of Birmingham and University Hospitals Birmingham NHS Foundation Trust, Birmingham, UK; 15 National Institute for Health Research Biomedical Research Centre at Moorfields Eye Hospital NHS Foundation Trust and University College London Institute of Ophthalmology, London, UK

**Keywords:** Artificial intelligence, Machine learning, Clinical trials, Randomised controlled trials, Research design, Research report, Guidelines, Checklist

## Abstract

**Background:**

The application of artificial intelligence (AI) in healthcare is an area of immense interest. The high profile of ‘AI in health’ means that there are unusually strong drivers to accelerate the introduction and implementation of innovative AI interventions, which may not be supported by the available evidence, and for which the usual systems of appraisal may not yet be sufficient.

**Main text:**

We are beginning to see the emergence of randomised clinical trials evaluating AI interventions in real-world settings. It is imperative that these studies are conducted and reported to the highest standards to enable effective evaluation because they will potentially be a key part of the evidence that is used when deciding whether an AI intervention is sufficiently safe and effective to be approved and commissioned. Minimum reporting guidelines for clinical trial protocols and reports have been instrumental in improving the quality of clinical trials and promoting completeness and transparency of reporting for the evaluation of new health interventions. The current guidelines—SPIRIT and CONSORT—are suited to traditional health interventions but research has revealed that they do not adequately address potential sources of bias specific to AI systems. Examples of elements that require specific reporting include algorithm version and the procedure for acquiring input data. In response, the SPIRIT-AI and CONSORT-AI guidelines were developed by a multidisciplinary group of international experts using a consensus building methodological process. The extensions include a number of new items that should be reported in addition to the core items. Each item, where possible, was informed by challenges identified in existing studies of AI systems in health settings.

**Conclusion:**

The SPIRIT-AI and CONSORT-AI guidelines provide the first international standards for clinical trials of AI systems. The guidelines are designed to ensure complete and transparent reporting of clinical trial protocols and reports involving AI interventions and have the potential to improve the quality of these clinical trials through improvements in their design and delivery. Their use will help to efficiently identify the safest and most effective AI interventions and commission them with confidence for the benefit of patients and the public.

## Background

Artificial intelligence (AI) is an area of immense and increasing interest within medicine. Developments in machine learning (ML) techniques, such as deep learning, and their application to data-rich problems, such as medical imaging, have highlighted several potential healthcare applications. Examples are wide ranging and include AI interventions for screening and triage [1–3], diagnosis [4–6], prognostication [7, 8], decision support [9], and treatment recommendation [10]. The high profile of ‘AI in health’ means that there are unusually strong drivers to accelerate the introduction and implementation of these innovative interventions, which may not be supported by the available evidence, and for which the usual systems of appraisal may not yet be sufficient.

## Main text

### The evidence gap for AI health interventions

Evidence that an AI intervention improves patient outcomes and is cost-effective is a prerequisite to implementation if the ultimate goal is to bring benefits to patients and society. However, in most cases, existing evidence for AI consists of in silico, early-phase, validation experiments. The outcome is mostly diagnostic or predictive accuracy and the comparator is often poorly reflective of real-world standards. These initial experiments provide early evidence of potential efficacy but, critically, they are not prospective and do not evaluate patient outcomes or provide evidence of cost-effectiveness.

The strongest evidence for the safety and efficacy of an intervention requires evaluation in the context of one or more randomised clinical trials [11]. This is true for all interventions, including those involving AI systems. Although most AI interventions in health have not yet been evaluated in clinical trials, this is likely to be an area of rapid expansion as the field matures, and as policy makers become clearer as to the evidence they require. These studies should place AI interventions within their intended clinical setting, consider patient outcomes as the primary endpoint, and consider potentially deleterious downstream consequences. Crucially, the evidence from these studies should be, like all trials, conducted and reported to the highest standard to enable effective evaluation, because they will potentially be a key part of the evidence that regulators, payers, and policy makers use when deciding whether an AI intervention is sufficiently safe and effective to be approved and commissioned.

### Complete and transparent reporting of clinical trial protocols and reports

The critical appraisal of clinical trials is an essential part of evidence-based practice where the quality of research and thereby the trustworthiness of its results are carefully and systematically evaluated [12]. Reviewers are able to assess the quality, value, and relevance of a clinical trial by considering the way it was designed, conducted, and analysed and by evaluating its internal and external validity. This process supports relevant stakeholders when making considered decisions about whether or not an intervention should be approved and commissioned.

The critical appraisal of clinical trial protocols is equally important in enabling readers and reviewers to evaluate proposed investigative plans. Reviewers of trial protocols can ensure investigators design clinical trials that should yield valid results in an ethically sound way. As a shared reference point, it also enables reviewers of clinical trial reports to ensure that investigators did what they intended on doing.

Critical appraisal is contingent on clear and comprehensive reporting. Reviewers cannot evaluate a clinical trial protocol unless investigators explain exactly what they intend on doing. Similarly, reviewers cannot evaluate a clinical trial unless investigators explain exactly what they did. This highlights two important characteristics of clinical trial protocols and reports: completeness and transparency of reporting.

The SPIRIT 2013 [13] (Standard Protocol Items: Recommendations for Interventional Trials) and CONSORT 2010 [14] (Consolidated Standards of Reporting Trials) statements are minimum reporting guidelines for clinical trial protocols and completed trials, respectively. The endorsement of these guidelines by the International Committee of Medical Journal Editors [15] as well as medical journals that require authors to comply by them at the point of submission has been instrumental in promoting completeness and transparency for the effective evaluation of new health interventions [16].

### The SPIRIT-AI and CONSORT-AI guidelines

Systematic reviews of existing clinical trials evaluating AI interventions have highlighted gaps in their reporting, and it has been recognised that current reporting guidelines do not adequately address potential sources of bias specific to AI systems [17, 18]. Examples of elements that require detailed and specific reporting include, but are not limited to, the algorithm version, the procedure for acquiring the input data, and the criteria for inclusion at the level of the input data in addition to the level of participants. For instance, in a clinical trial evaluating an AI system for diagnosing knee osteoarthritis using knee radiographs, authors must specify which version of the AI system was used and state whether this changed throughout the course of the trial; describe how knee radiographs were acquired, selected, and pre-processed before analysis by the AI system; and report the eligibility criteria at both the level of participants, such as patient age, and input data, such as knee radiograph image quality. Detailed and specific reporting of the input data criteria separately to the participant criteria is especially important as it enables evaluators to differentiate between those AI interventions that only work in ideal conditions and those that are more robust and suitable for real-world settings.

The risk of an AI intervention being approved and commissioned based on incomplete information highlights the need for AI-specific reporting guidance. To address this, the SPIRIT-AI and CONSORT-AI Steering Group announced in October 2019 an initiative to develop evidence-based extensions for clinical trial protocols and reports involving AI interventions [19]. The SPIRIT-AI [20–22] and CONSORT-AI [23–25] guidelines have since been developed in accordance with the EQUATOR (Enhancing the Quality and Transparency of Health Research) Network recommendations. The guidelines were developed using a Delphi methodology with an international multidisciplinary consortium. The consensus process involved relevant stakeholders with expertise in the application of AI in health and key users of the technology. Stakeholders included clinicians, computer scientists, experts in law and ethics, funders, health informaticists, industry partners, journal editors, methodologists, patients, policy makers, regulators, and statisticians.

The SPIRIT-AI [20–22] and CONSORT-AI [23–25] guidelines include 15 and 14 new items, respectively, that should be routinely reported in addition to the core SPIRIT 2013 [13] and CONSORT 2010 [14] items. For example, the new guidelines recommend that investigators should provide clear descriptions of the AI intervention, including instructions and skills required for use, the study setting in which the AI intervention is integrated, the handling of inputs and outputs of the AI intervention, the human-AI interaction, and the analysis of error cases. Each item, where possible, was informed by challenges identified in existing studies of AI systems in health settings.

The SPIRIT-AI [20–22] and CONSORT-AI [23–25] guidelines have the potential to improve the quality of clinical trials of AI systems in health, through improvements in design and delivery, and the completeness and transparency of their reporting. It is, however, important to appreciate the context in which these guidelines sit. First, the new items within SPIRIT-AI [20–22] and CONSORT-AI [23–25] are all extensions or elaborations rather than substitutes for the core items. Core considerations addressed by SPIRIT 2013 [13] and CONSORT 2010 [14] remain important in all clinical trial protocols and reports, regardless of the intervention itself. In addition, depending on the trial design and outcomes, other SPIRIT or CONSORT guidelines may be relevant [26, 27].

Second, SPIRIT-AI [20–22] and CONSORT-AI [23–25] are specific to clinical trials, whereas it should be recognised that most current evaluations of AI systems are some form of diagnostic accuracy or prognostic model study. AI-specific guidelines that will address such studies, STARD-AI [28] (Standards for Reporting Diagnostic Accuracy Studies – Artificial Intelligence) and TRIPOD-AI [29] (Transparent Reporting of a Multivariable Prediction Model for Individual Prognosis or Diagnosis – Artificial Intelligence), are currently under development. Whilst there are likely to be common elements between these AI extensions, investigators reporting should use, and reviewers appraising should receive, the most suitable guideline available which considers both the study design *and* the type of intervention.

Third, the SPIRIT-AI [20–22] and CONSORT-AI [23–25] guidelines will evolve to keep pace with this fast-moving field. The dearth of clinical trials involving AI interventions to date means that discussions that took place and decisions that were made during the development of these guidelines were not always supported by ‘real life’ lessons from the literature. Additionally, the recommendations are most relevant to the current context and contemporaneous challenges. The extensions proactively rather than reactively address reporting issues in this rapidly evolving field and, naturally, newer and more nuanced versions will be necessary as the field continues to evolve.

For example, SPIRIT-AI [20–22] and CONSORT-AI [23–25] were mostly informed by current applications of AI, mainly focussing on disease detection and diagnosis, and will need updating as additional applications such as those that utilise AI as therapy begin to emerge. Similarly, current extensions do not yet address AI systems involving ‘adaptive’ algorithms. The performance of these types of AI systems—which continue to ‘learn’ as they are updated or tuned on new training data—can change over time. This is unlikely to pose a problem at present because these AI systems are still at an early stage in their development but will be an important issue to address in future iterations.

## Conclusion

The SPIRIT-AI [20–22] and CONSORT-AI [23–25] guidelines—co-published in *Nature Medicine*, *The BMJ*, and *The Lancet Digital Health* in September 2020—provide the first international standards for clinical trials of AI systems in health. The extensions are designed to ensure complete and transparent reporting and enable effective evaluation of clinical trial protocols and reports involving AI interventions. They have been developed by key stakeholders from across sectors with widespread support and representation. As we now look to their implementation, we are delighted that leading medical journals including *Trials* are endorsing these guidelines, and their extensions, and encouraging their widespread adoption to support the design, delivery, and reporting of clinical trials of AI systems in health. It is only through this process that we will be able to adequately evaluate AI interventions, and enable safe and effective systems to be deployed with confidence for the benefit of patients and the public.

## Data Availability

Not applicable.

## Abbreviations

AI: Artificial intelligence
EQUATOR: Enhancing the Quality and Transparency of Health Research
CONSORT: Consolidated Standards of Reporting Trials
CONSORT-AI: Consolidated Standards of Reporting Trials - Artificial Intelligence
ML: Machine learning
SPIRIT: Standard Protocol Items: Recommendations for Interventional Trials
SPIRIT-AI: Standard Protocol Items: Recommendations for Interventional Trials - Artificial Intelligence
STARD-AI: Standards for Reporting Diagnostic Accuracy Studies - Artificial Intelligence
TRIPOD-AI: Transparent Reporting of a Multivariable Prediction Model for Individual Prognosis or Diagnosis - Artificial Intelligence
